# Engineering of Doxorubicin-Encapsulating and TRAIL-Conjugated Poly(RGD) Proteinoid Nanocapsules for Drug Delivery Applications

**DOI:** 10.3390/polym12122996

**Published:** 2020-12-16

**Authors:** Elad Hadad, Safra Rudnick-Glick, Ella Itzhaki, Matan Y. Avivi, Igor Grinberg, Yuval Elias, Shlomo Margel

**Affiliations:** 1 Department of Chemistry, Institute of Nanotechnology & Advanced Materials, Bar-Ilan University, Ramat Gan 5290002, Israel; elad.hadad@yahoo.com (E.H.); safrar@gmail.com (S.R.-G.); Elaeli3543@gmail.com (E.I.); grinbergigori97@yandex.com (I.G.); yuvalel@biu.ac.il (Y.E.); 2 The Mina and Everard Goodman Faculty of Life Sciences, Institute of Nanotechnology & Advanced Materials, Bar-Ilan University, Ramat Gan 5290002, Israel; matanavivi88@gmail.com

**Keywords:** RGD, proteinoid, doxorubicin, TRAIL

## Abstract

Proteinoids are non-toxic biodegradable polymers prepared by thermal step-growth polymerization of amino acids. Here, P(RGD) proteinoids and proteinoid nanocapsules (NCs) based on D-arginine, glycine, and L-aspartic acid were synthesized and characterized for targeted tumor therapy. Doxorubicin (Dox), a chemotherapeutic drug used for treatment of a wide range of cancers, known for its adverse side effects, was encapsulated during self-assembly to form Dox/P(RGD) NCs. In addition, tumor necrosis factor-related apoptosis-inducing ligand (TRAIL), which can initiate apoptosis in most tumor cells but undergoes fast enzyme degradation, was stabilized by covalent conjugation to hollow P(RGD) NCs. The effect of polyethylene glycol (PEG) conjugation was also studied. Cytotoxicity tests on CAOV-3 ovarian cancer cells demonstrated that Dox/P(RGD) and TRAIL-P(RGD) NCs were as effective as free Dox and TRAIL with cell viability of 2% and 10%, respectively, while PEGylated NCs were less effective. Drug-bearing P(RGD) NCs offer controlled release with reduced side effects for improved therapy.

## 1. Introduction

Ovarian cancer is a very common cancer with high mortality due to poor detection at early stage [1,2]. The risk of developing ovarian cancer is one in 75 with five-year survival after late detection of only about 30%, considerably lower than after early detection [3].

Doxorubicin hydrochloride (Dox), a chemotherapeutic drug, which intercalates with DNA, is used for a wide range of cancer treatments [4,5], however it is associated with severe side effects, e.g., cardiac damage, vomiting, bone marrow suppression, tissue damage at the site of injection, and myelosuppression, which prevent it from being the prominent first choice [6,7]. Tumor necrosis factor (TNF)-related apoptosis-inducing ligand (TRAIL), a protein considered as a promising candidate for treatment owing to its selective apoptotic effect on cancer cells [8,9], is not therapeutically effective, as IV administration leads to insufficient amount at the target site due to rapid degradation in vivo. Previous studies showed that the conjugation of TRAIL or other bioactive proteins or peptides (e.g., thrombin, NGF, BDNF, bFGF, or tPA) to nanoparticles stabilizes them against proteolytic enzymes and inhibitors, resulting in a significant improvement in their in vivo activity [10,11,12,13,14].

RGD, a tripeptide composed of arginine (R), glycine (G), and aspartic acid (D), was initially discovered in 1985 by Pierschbacher et al. as the active component of fibronectin protein [15]. RGD is attracted to areas of angiogenesis due to its high affinity to αvβ3 integrin, which is overexpressed in many cancer cells and highly up-regulated on the surface of growing tumor blood vessels [16,17,18], leading to the development of integrin-targeted nano-drugs for imaging and treatment of tumors. Both linear and cyclic RGD peptides were used for conjugation of RGD to nanoparticles for targeted drug delivery [19].

Proteinoids—polymers prepared by thermal step-growth polymerization of natural or synthetic amino acids discovered by Fox [20,21,22,23,24,25]—are considered to be non-immunogenic, biodegradable, and non-toxic [26,27], and may therefore be used for drug delivery. Here, P(R^D^GD) proteinoids composed of D-arginine and L-aspartic acid were synthesized with the intention of randomly achieving the R^D^GD sequence in the proteinoid backbone. Previous work illustrated encapsulation of various amphiphiles, e.g., dyes, [28,29,30] vitamin A [31], and Ouxin [32], for diagnostic imaging, reduction-responsive vehicle, cosmetics, and agriculture. P(R^D^GD) nanocapsules (NCs) exhibit higher selectivity toward angiogenesis sites compared to other configurations of amino acids such as P(R^D^GD^D^), which includes D-aspartic acid [33].

Proteinoids are formed in the absence of catalyst or solvent by heating certain amino acids in an inert atmosphere [34,35]. Aspartic acid serves as a solvent for the other amino acid monomers and is condensed upon heating through cyclization into 2,2′-(3,6-dioxopiperazine-2,5-diyl)diacetic acid [36]. This initiates polymerization with the other amino acids, forming a proteinoid polymer [20]. Previous study in our lab demonstrated via NMR that the RGD content in P(RGD) is about 11% [33].

NCs formed via self-assembly of proteinoids in an aqueous continuous phase can encapsulate chemotherapeutic agents such as Dox [37]. Self-assembly occurs due to the many functional groups in the polymer backbone. Upon precipitatation, hydrophobic residues such as alkyl groups form a hydrophobic core, while carboxylic groups on the particle surface form hydrogen bonds [36]. Furthermore, the terminal amines of the proteinoids on the surface can be utilized for conjugation. Here, TRAIL was conjugated to hollow P(RGD) NCs, forming another potential therapeutic vehicle.

Previous studies showed that PEGylated drugs reduce penetration rates into cells [36] and induce formation of antibodies [38]. Yet, PEGylation still has a major role in drug delivery [36], for example in evading phagocytosis [39] and increasing blood circulation [40]. Dox-encapsulating Dox/P(RGD) and TRAIL-conjugated TRAIL-P(RGD NCs were PEGylated to compare their penetration rate with non-PEGylated NCs.

## 2. Materials and Methods

### 2.1. Materials

NHS-PEG (3500 MW) maleimide ester was purchased from Advanced BioChemicals (Lawrenceville, GA, USA). The following chemicals were obtained from Sigma and used without further purification: D-arginine, L-arginine, glycine, NaCl, bicarbonate buffer (BB, 0.1 M), 1-ethyl-3-(3-dimethylaminopropyl) carbodiimide (EDC), *N*-hydroxysulfosuccinimide (sulfo-NHS) and 2-morpholinoethanesulfonic acid (MES, pH = 6). Doxorubicin HCl was purchased from Tzamal D-Chem laboratories (Petah-Tikva, Israel), and human TRAIL was acquired from Peprotech (Rehovot, Israel). CAOV-3 ovarian cell lines were purchased from ATCC (Manassas, VA, USA) and their medium was purchased from Lonza (Basel, Switzerland). PBS (0.01 M) and XTT kits were obtained from Biological Industries (Beit Haemek, Israel). Double distilled water (DDW) was purified by passing deionized water through an Elgastat Spectrum reverse osmosis system (Elga Ltd., High Wycombe, UK). An ELISA kit for TRAIL was purchased from Biotest Ltd. (Beit Haemek, Israel).

### 2.2. Synthesis of P(RGD) Proteinoid by Step-Growth Polymerization Mechanism

Mixtures of amino acids (5 g, 1.666 g each of D-arginine, glycine, and L-aspartic acid) in a three-neck flask were heated under N_2_ atmosphere on a heating mantle to 180 °C until full dissolution. The mixture was stirred by a mechanical stirrer at 250 rpm for 20 min, producing a highly viscous yellow brown paste. The paste was allowed to cool to room temperature (RT) and harden until a glassy mass was formed. After cooling, the residual material was extracted using 30 mL of distilled water and lyophilized to yield the crude P(RGD) proteinoid.

### 2.3. Proteinoid Characterization and Analysis

The molecular weights and polydispersity index of the proteinoid polymer were determined by gel permeation chromatography (GPC) consisting of a Waters Spectra Series P100 isocratic HPLC pump with ERMA ERC-7510 refractive index detector and Rheodyne injection valve (Coatati, CA, USA) with a 20 µL loop (Waters, Milford, MA, USA). The sample was dissolved with super-pure HPLC water (Sigma) through linear BioSep SEC-s3000 column (Phenomenex, Torrence, CA, USA) at a flow rate of 1 mL/min. The molecular weight of the proteinoid was determined relative to poly(ethylene glycol) (PEG) standards (Polymer Standards Service-USA, Silver Spring, MD, USA) with a molecular weight range of 0.10−450 kDa, bovine plasma fibrinogen (340 kDa), and human serum albumin (67 kDa) using Clarity chromatography software (DataApex, Prague, Czech Republic).

The absorption spectrum of the proteinoid was obtained by using a Cary 100 UV-Visible (UV-Vis) spectrophotometer (Agilent Technologies, Santa Clara, CA, USA). Fourier transform infrared (FTIR) measurement of the proteinoid was performed by the Attenuated Total Reflectance (ATR) technique using a Bruker Alpha FTIR QuickSnapTM sampling module equipped with a Platinum ATR diamond module (Bruker Optik GmbH, Ettlingen, Germany).

### 2.4. Preparation of Hollow P(RGD) NCs

P(RGD) NCs were prepared by a self-assembly process. Briefly, 50 mg of dried proteinoid in 0.3 mL PBS buffer (0.15 M) with pH 7.4 was added to 5 mL of a 0.01 mM NaCl aqueous solution. The mixture was stirred at 250 rpm and heated to 80 °C until the proteinoid dissolved completely. After 30 min, the heating was stopped and the solution was allowed to cool slowly to RT, producing the proteinoid NCs.

### 2.5. Preparation of Dox/P(RGD) NCs

Dox-encapsulating Dox/P(RGD) NCs were prepared by self-assembly, as described above. Briefly, proteinoid (50 mg) dispersed in 0.5 mL PBS buffer was added to 0.01 mM NaCl solution (5 mL), and the mixture was stirred at 250 rpm and heated to 80 °C until complete dissolution. 1.25 mg of Dox (2.5% *w*/*w* relative to the proteinoid weight) was dissolved in heated NaCl solution (0.1 mL, 80 °C) and immediately added to the heated proteinoid dispersion. After a few minutes (approximately 5 min), the mixture was left to slowly cool to RT. The Dox/P(RGD) aqueous dispersions underwent extensive dialysis to remove excess Dox using a cellulose membrane (100 KDa MW cutoff) against DDW.

### 2.6. PEGylation of Dox/P(RGD) NCs

PEGylated hollow and Dox/P(RGD) NCs were prepared by coupling free primary amine groups of the hollow and Dox/P(RGD) NCs with NHS-PEG (3500 MW) maleimide ester. Briefly, hollow P(RGD) and Dox/P(RGD) NCs (5 mg/mL) were dispersed in PBS (5 mL, 0.1 M, pH = 7.4). NHS-PEG maleimide ester (0.05 mg) dissolved in 0.1 mL DDW was added to the NC dispersions in PBS. The reaction was stirred for 1 h at RT to form PEGylated maleimide derivatized P(RGD) and Dox/P(RGD) NCs dispersed in PBS. The pH was then raised to 8.3 by addition of BB (0.1 M) to the dispersions. Glycine (10 µg) dissolved in BB was added, and the derivatized NC dispersion was shaken at RT for 1 h in order to block residual maleimide groups. The obtained PEGylated hollow and Dox/P(RGD) NCs were then washed from undesired reagents by extensive dialysis (10 kDa cutoff) against DDW at 4 °C, followed by filtration through a 0.2 µm syringe filter.

### 2.7. Indirect Conjugation of TRAIL to P(RGD) NCs

TRAIL was covalently conjugated to maleimide-PEG hollow P(RGD) NCs by Michael addition of the primary amino groups of TRAIL to activated double bonds of the maleimide derivatized NCs. Briefly, aqueous BB solution of TRAIL (50 µg in 0.3 mL, 0.1 M) was added to aqueous BB dispersions of PEGylated maleimide P(RGD) NCs (2.5 mg/mL, pH = 8.3). The obtained bicarbonate aqueous dispersions were shaken at 4 °C overnight. The blocking of residual double bonds was accomplished by adding 10 µg of glycine and shaking for an additional 1 h. The obtained TRAIL-P(RGD) NCs were washed from undesired reagents by extensive dialysis, followed by filtration, as described above.

### 2.8. Direct Conjugation of TRAIL to P(RGD) NCs

TRAIL was covalently conjugated to hollow P(RGD) NCs through carbodiimide activation of carboxylate groups on the NC surface [41]. Briefly, sulfo-NHS and EDC (1 mg each) were dissolved separately in 1 mL of 0.1 M MES buffer. Sulfo-NHS (50 μL, 1 mg/mL) and EDC (20 μL, 1 mg/mL) solutions were added to an aqueous dispersion of hollow P(RGD) NCs (2.5 mg/mL) and shaken at RT for 15 min, TRAIL (50 μg) dissolved in bicarbonate buffer (50 µL, 0.1 M) was added, and the dispersion was shaken at 4 °C overnight. The obtained TRAIL-P(RGD) NCs were washed from undesired reagents by extensive dialysis and filtration, as described above.

### 2.9. Diameter and Size Distribution Measurements

The dry diameter and size distribution of the hollow P(RGD), Dox/P(RGD), TRAIL-P(RGD), and PEGylated Dox/P(RGD) and TRAIL-P(RGD) NCs were measured with high resolution scanning electron microscopy (HR-SEM) and analyzed by ImageJ software, an open source Java image processing program, as described previously [33].

The hydrodynamic diameters and size distribution of the NPs were characterized by dynamic light scattering (DLS, Vasco 2, Cordouan Technologies SAS, Pessac, France). Briefly, a few drops of dispersed PEGylated or non-PEGylated Dox/P(RGD) NCs were added to a sample cell and analyzed at 25 °C at pH 7.4.

### 2.10. ζ-Potential Measurements

The P(RGD) NC surface potential was measured in aqueous dispersion at pH 7.4 at a concentration of 1 mg/mL using a Zetasizer 3000 HSa ζ-potential analyzer (Malvern Instruments Company, Malvern, UK).

### 2.11. Quantification of Conjugated TRAIL

The concentration of conjugated TRAIL was determined by enzyme-linked immunosorbent assay (ELISA) according to the manufacturer instructions (R&D Systems, Quantikine, Human TRAIL/TNFSF10) [38].

### 2.12. HPLC Analysis

HPLC analysis was performed using a Hitachi LaChrom Elite system (Hitachi, Tokyo, Japan) equipped with a fluorescence detector, column oven, autosampler, quaternary pump, and 100 μL sample loop. The chromatographic separation was performed at 30 °C using an ACE 5 C-18 column (5 µm particle size, 250 × 4.6 mm) under gradient elution conditions. Mobile phases consisting of water-trifluoroacetic acid (999:1, *v*/*v*) (A) and acetonitrile-trifluoroacetic acid (999:1, *v*/*v*) (B) were filtered through a membrane filter (0.22 μm). Gradient elution started at 3% B, increased linearly to 30% B for 3 min, and then increased linearly to 50% B until 10 min and stayed isocratic for the next 5 min. For column equilibrium, the gradient was set back to 3% B, and the system was allowed to equilibrate until 20 min at a flow rate of 1.0 mL/min. The injection volume was 5 μL, and the column elution was monitored at an excitation of 490 nm and emission of 590 nm. Chromatogram peak integration and area calculation were performed using EZChrom Elite Software. The retention times were 8.17 ± 0.01 min for the standard (*n* = 4) and 8.14 min for both samples. Dox was extracted from Dox/P(RGD) NCs dispersed in water/ethanol 1:1 (5 mg/mL) via sonication in a bath sonicator for 5 min at RT, as described previously. Encapsulated Dox concentration was determined according to a standard curve of known analyte concentrations.

### 2.13. Optical Characterization of Dox and Dox/P(RGD) NCs

Free and encapsulated Dox were characterized at excitation and emission wavelengths of 470 nm and 590 nm, respectively, using a Cary Eclipse spectrophotometer (Agilent Technologies Inc.).

### 2.14. Photostability of Free and Encapsulated Dox

Aqueous solutions/dispersions of Dox and Dox/P(RGD) NCs were prepared to give similar fluorescence intensity. Intensities were measured with λex set at 490 nm and λem of 590 nm. Each sample was illuminated continuously with a xenon lamp, and the fluorescence intensity was measured over a period of 5 min by a Synergy fluorescence spectrophotometer (Agilent Technologies Inc.). Intensity values were normalized for comparison.

### 2.15. Drug Release Model

Aqueous dispersions of Dox/P(RGD) NCs (300 µL, 10 mg/mL) were incubated with PBS buffer (0.15 M, 4.5 mL) with pH = 7.4, exclusively or with a mixture of human serum (0.5 mL) and PBS buffer (4 mL) at 37 °C, giving a final NC concentration of 1 mg/mL in a total volume of 5 mL. Samples were collected at several time periods, and the released Dox was measured using a Cary 100 UV-Vis spectrophotometer (Agilent Technologies Inc.).

### 2.16. In Vitro XTT Cell Viability Assay

An XTT assay was performed to determine the viability of CAOV-3 human ovarian cancer cells after treatment with free drug or drug-bearing NCs. Cells were seeded in a 96 well plate at a density of 10^4^ cells/well in culture medium (100 μL) and grown in a humidified 5% CO_2_ atmosphere at 37 °C. After 48 h, free drug or NCs dispersed in DDW were diluted in culture medium and added to the cells, giving a final concentration of 0.5 mg/mL per well. After incubation for 48 h, 72 h, and 96 h at 37 °C, 50 μL XTT solution was added to each well according to the kit manufacturer’s instructions. Absorbance was read at 490 nm, and cell viability was determined using the procedure recommended in the manufacturer’s protocol [42].

## 3. Results and Discussion

### 3.1. Synthesis and Characterization of P(RGD) Proteinoid Nanocapsules (NCs)

P(RGD) nanocapsules (NCs) for targeted delivery were synthesized as described in Ref [36]. Briefly, mixtures of amino acids (5 g total weight) were heated under nitrogen to 180 °C and stirred at 250 rpm for 20 min in a three-neck flask until full dissolution; the resultant yellowish to brownish paste was allowed to cool to RT and harden to from a glassy mass. The residual material was extracted by distilled water (30 mL) and lyophilized. According to GPC analysis, the as-synthesized proteinoid had an average molecular weight of 67,660 Da, number average molecular weight of 67,640 Da, and molecular weight at the peak of 66,465 Da, as described in Ref [36] (see Table 1 therein). Further characterization of the proteinoids by ATR and UV absorbance is included in the Supplementary Material (see Supplementary Figure S1).

### 3.2. Synthesis and Characterization of P(RGD) NCs

**Figure 1:**
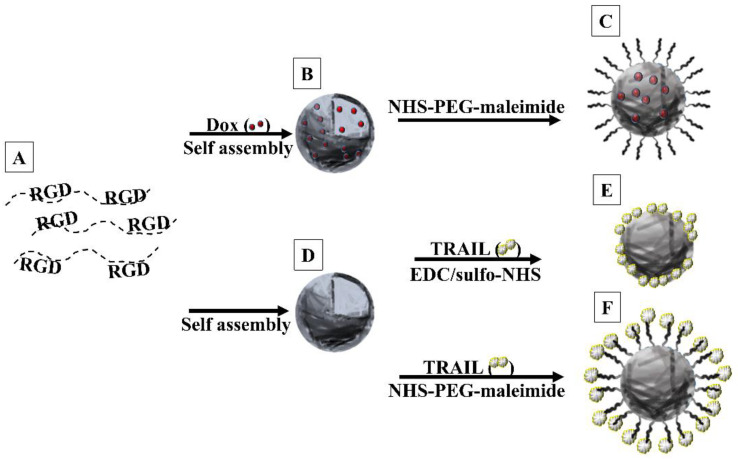
Scheme illustrating formation of various P(RGD) NCs. (**A**) RGD proteinoid polymers were self-assembled (**B**) with Dox and subsequently (**C**) PEGylated, or (**D**) without Dox and conjugated to TRAIL either (**E**) directly or (**F**) indirectly via PEG (3500 MW).

The synthesis scheme of hollow and Dox-encapsulating and TRAIL-conjugated P(RGD) NCs is demonstrated in Figure 1. Hollow NCs (Figure 1D) were formed via a self-assembly process of the soluble P(RGD) proteinoid polymer (Figure 1A) through precipitation in an aqueous solution by slow cooling to RT of the soluble P(RGD) at 80 °C. Incorporation of Dox in the self-assembly process resulted in formation of Dox/P(RGD) NCs (Figure 1B). PEGylation was performed with NHS-PEG (3500 MW) maleimide ester by binding surface primary amino groups of the NCs to the PEG spacer via its NHS functional groups at physiological pH (7.4, Figure 1C). If necessary, residual double bonds belonging to the maleimide were quenched with glycine in bicarbonate buffer (pH 8.3). Previous work in our lab illustrated that conjugation of TRAIL to nanoparticles increases its stability and reduces its enzymatic degradation [9]. Therefore, TRAIL was covalently conjugated to the surface of the NCs. In order to study the optimal conditions for conjugation, TRAIL was bonded to the NCs by two different methods: direct binding via sulfo-NHS/carbodiimide activation of surface carboxylate groups of the NC to the primary amino groups of TRAIL (Figure 1E) [43] and indirect binding through a PEG spacer via Michael addition to the surface maleimide groups (Figure 1F) [44]. The optimal TRAIL conjugation method was detemined by charectrization of the NCs. SEM was used to confirm that the NC size and shape did not alter following conjugation. The concentration of bound TRAIL was determined by ELISA.

**Figure 2:**
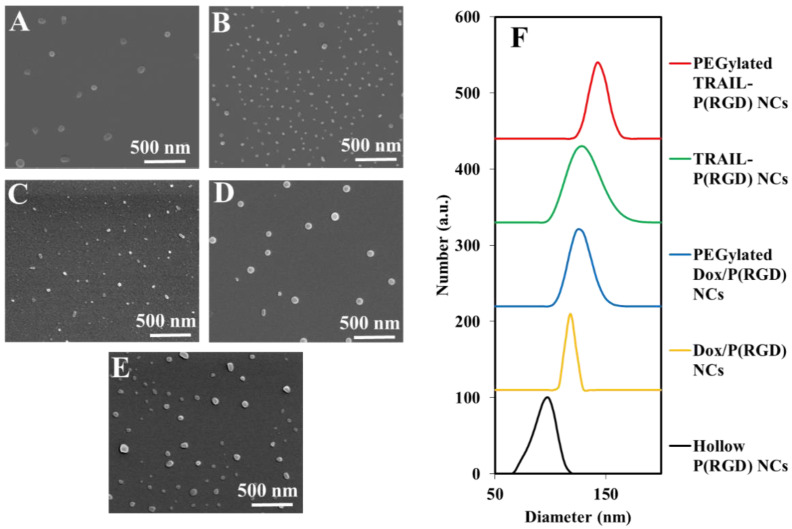
SEM images of P(RGD) NCs. (**A**) Hollow, (**B**) PEGylated Dox-loaded, (**C**) Dox-loaded, (**D**) PEGylated TRAIL-conjugated, and (**E**) TRAIL-conjugated NCs. (**F**) Diameter distributions measured by DLS.

Figure 2 shows SEM images of hollow and drug-bearing P(RGD) NCs, and Table 1 summarizes the diameters and size distributions. It is interesting to see that encapsulation of the hydrophobic Dox led to a significant decrease in diameter from 84 ± 16 to 44 ± 16 nm; this apparent shrinking of the capsules is in accord with a recent report by Abd-Allah who showed that the particle size can decrease, depending on the encapsulated molecule [45].

On the other hand, as expected, the dry diameter did not significantly change following PEGylation of the Dox/P(RGD) NCs. Rahme et al. reported that the difference in layer thickness of PEG with MW in the range of 750–5000 is ~0.42 nm. This supports the results in Table 1 where no significant change in diameter is observed [46]. The table indicates that TRAIL conjugation and PEGylation increased the diameter only slightly, from 84 ± 6 to 86 ± 10 and 90 ± 12 nm, respectively.

As expected, all drug-bearing NCs exhibited significantly larger hydrodynamic diameters (see Table 1). Following indirect PEGylation, the hydrodynamic diameter with Dox and TRAIL increased to 130 ± 17 nm and 146 ± 16 nm from 118 ± 7 nm and 134 ± 32 nm, respectively, as shown in Figure 2F. Zeta potential (ζ-potential) measurement at physiological pH (7.4) demonstrated a significant negative charge for the hollow and non-PEGylated drug-bearing P(RGD) NCs due to the carboxyl residue on the particle surface [36,37,47]. As observed recently [36], the elevated ζ-potential of the PEGylated NCs may indicate a successful conjugation of PEG to the surface residues of the P(RGD) NCs.

### 3.3. Loading Capacity of Dox and TRAIL in P(RGD) NCs

**Figure 3:**
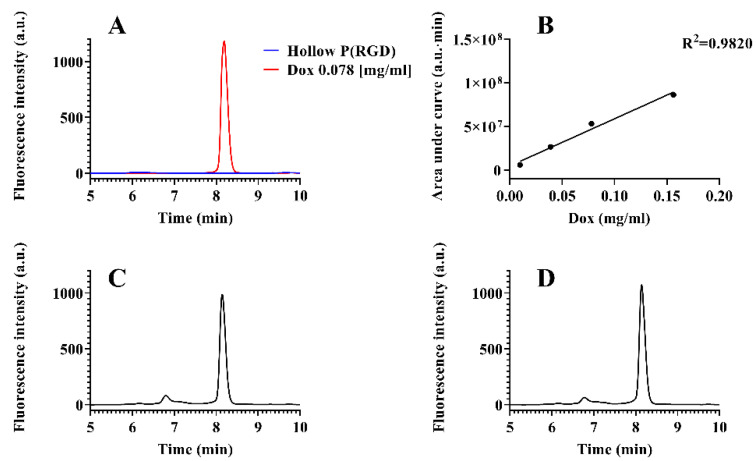
Determination of encapsulated Dox concentration in Dox/P(RGD) NCs. (**A**) Chromatograms of hollow P(RGD) NCs (blue) overlaid with a standard sample of 0.078 mg/mL Dox (Red). Dox was detected after 8.17 min. (**B**) Standard calibration curve of known Dox concentrations. Chromatograms of (**C**) Dox/P(RGD) and (**D**) PEGylated Dox/P(RGD) NCs, respectively. Dox was detected after 8.14 min in both samples as well as a small impurity at 6.8 min.

Doxorubicin (Dox) has maximum excitation and emission wavelengths of 470–490 nm and 590 nm, respectively, and can be selectively detected using fluorescence. Therefore, we used high-performance liquid chromatography (HPLC) with fluorescence detection to determine the amount of encapsulated Dox, as described in Section 2.12. Figure 3A exhibits chromatographic separation of hollow P(RGD) NCs and a standard sample of Dox (78 µg/mL). The standard sample showed a clear peak at retention time of 8.17 min, while no peaks where observed in the spectrum of the hollow P(RGD) NCs. A standard calibration curve was obtained according to known Dox concentrations, and the encapsulated drug concentration in the non-PEGylated and PEGylated Dox/P(RGD) NCs was determined, yielding 67 µg/mL and 74 µg/mL, respectively, as shown in Figure 3C,D. The small impurity at about 6.8 min is probably due to the encapsulation process. To exclude the possibility of Dox degradation during the encapsulation, a sample of degraded Dox was analyzed. In contrast to the tested NC samples, which showed a well-defined peak corresponding to undegraded Dox, the degraded Dox chromatogram displayed three major peaks with decreasing intensity (Supplementary Figure S2), suggesting that Dox remained intact during the encapsulation.

TRAIL was conjugated to hollow P(RGD) NCs via a PEG spacer and directly via carbodiimide conjugation. The concentration of TRAIL conjugated to the NC surface was determined using ELISA and calculated to be 150 ng/mL and 5 ng/mL for 1 mg/mL of PEGylated and non-PEGylated TRAIL-P(RGD) NCs, respectively. These results indicate, as expected, that the addition of a PEG spacer arm leads to a significant increase in the TRAIL binding capacity. Entrapment efficiency of about 60% was achieved for both Dox/P(RGD) NCs (see Table 2). TRAIL conjugation efficiency was much lower, i.e., 0.75% for the PEGylated NCs and only 0.025% for the TRAIL-P(RGD) NCs.

### 3.4. Optical Characterization of Free Dox and Dox/P(RGD) NCs

**Figure 4:**
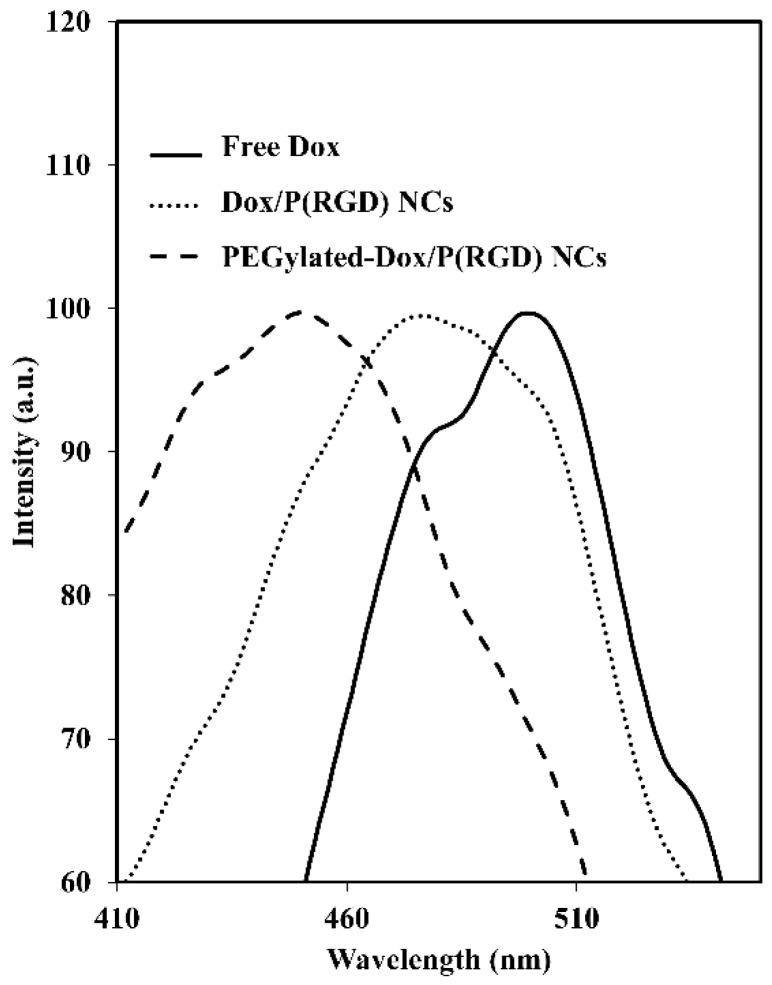
Excitation curves of free Dox (solid line) and non-PEGylated (dotted line) and PEGylated (dashed line) Dox/P(RGD) NCs.

Figure 4 exhibits the excitation peak at 498 nm of free Dox, and excitation peaks at 480 nm and 450 nm for the non-PEGylated and PEGylated Dox/P(RGD) NCs, respectively. This shift towards the left is described in the literature as a blue shift in the fluorescence [48]. This is most likely caused by the encapsulation within the small interior space of the NC, causing aggregation of the dye (Dox) molecules [49,50]. The increased blue shift probably results from the smaller interior due to the shielding of Dox by the PEG layer.

### 3.5. Photostability of Free Dox and Dox/P(RGD) NCs

**Figure 5:**
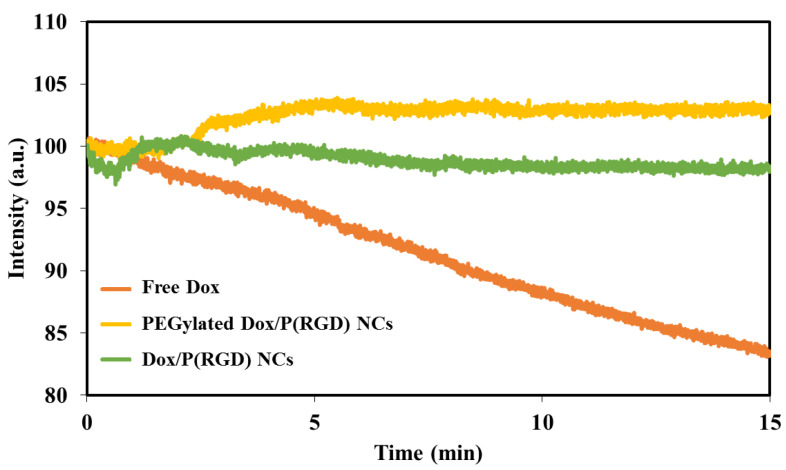
Photostability of free Dox (orange line) and non-PEGylated (green line) and PEGylated (yellow line) Dox/P(RGD) NCs. Illumination was performed continuously at 490 nm for a period of 5 min.

The encapsulation of a fluorophore was reported to increase the photostability, as reflected by reduced dye fluorescence degradation [49]. Here, illumination was performed continuously at 490 nm for a period of 15 min for free Dox and Dox/P(RGD) NCs. The fluorescence intensity of the Dox/P(RGD) NCs was not affected by the continuous illumination and remained at 98%, while the PEGylated Dox/P(RGD) NCs showed a slight increase to 102%. Free Dox fluorescence decreased by 17% (100% to 83%), as shown in Figure 5. This indicates that Dox is encapsulated within the NCs and is protected against oxidation, reducing agents, heat, illumination levels, and exposure time, which may decrease the fluorescence intensity [50,51].

### 3.6. Controlled Release of Dox from P(RGD) Nanocapsules

**Figure 6:**
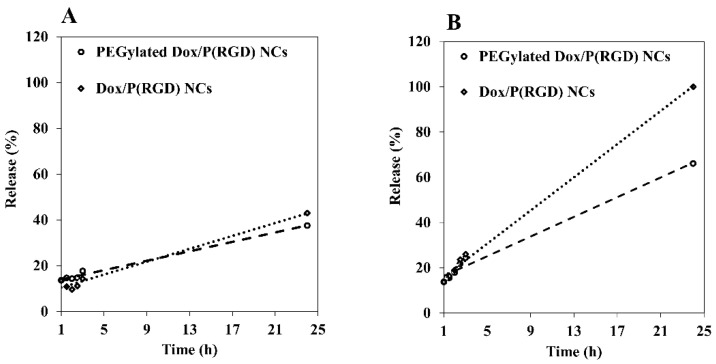
Relative release of non-PEGylated (dotted line) vs. PEGylated (dashed line) Dox/P(RGD) NCs in (**A**) PBS and (**B**) human serum over a 24 h incubation period at 30 °C.

A common method for studying in vitro drug release is by incubation in PBS or human serum [51]. Figure 6A shows the release profile of Dox from Dox/P(RGD) NCs dispersed in PBS. The drug release pattern depends on a variety of factors, including the drug and polymer structures. The effects of PEGylation and of the media on the release rate were investigated. Dox was released at a rapid rate after a period of 24 h when dispersed in serum, however the release rate was very slow in PBS dispersion, as demonstrated in Figure 6B. The highest release rates were obtained within 24 h, with a maximum release in PBS of around 40%. In human serum, the sustained release of Dox from both non-PEGylated and PEGylated NCs is clearly evident following 24 h, with the highest release rate found for Dox/P(RGD). After 3 h of incubation, the rate was only 26% but increased significantly after 24 h, reaching 99%. For the PEGylated NCs, similar release of about 26% was obtained after 3 h, however only 60% was released after 24 h. This difference in Dox release can be explained by the presence of PEG, which acted as an additional protective layer, decreasing the amount of exposed degradable bonds of the proteinoid NCs and impeding their biodegradation.

### 3.7. Cytotoxicity of Drugs and NCs

**Figure 7:**
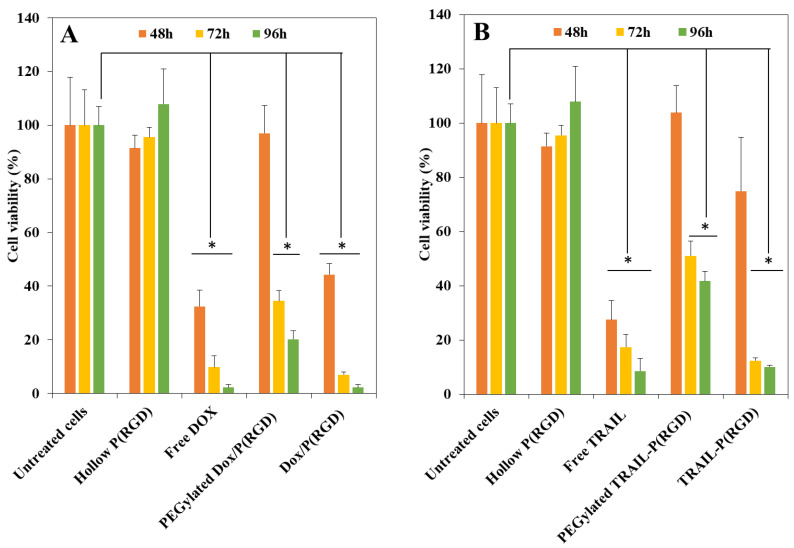
Cell viability of untreated and CAOV-3 treated cells after 48 h (orange), 72 h (yellow) and 96 h (green) with hollow P(RGD) NCs as control of (**A**) free Dox and Dox/P(RGD) NCs and (**B**) free TRAIL and TRAIL-P(RGD) NCs. * *t* test *p* < 0.05, error bars represent standard deviation.

The therapeutic activity of the drugs and NCs was investigated in ovarian cancer cells. CAOV-3 cells were treated for 48 h, 72 h, and 96 h with free drug or hollow/drug-loaded/conjugated P(RGD) NCs (0.7 µg/mL). Free Dox (0.7 µg/mL) and TRAIL (0.5 µg/mL) and untreated cells were used as controls. Figure 7A demonstrates the cell viabilities following 48 h, 72 h, and 96 h of treatment with Dox. The hollow NCs did not exhibit any toxicity throughout the 96 h time period; moreover, viability was increased beyond untreated cells, probably due to uptake of P(RGD) NCs as a nutrient, while viability after treatment with free Dox was low—32% after 48 h, which decreased to 9% and 2%.

The PEGylated Dox/P(RGD) NCs did not exhibit significant toxicity after 48 h of treatment; only 34% viability was observed after 72 h, which reduced to 20% after 96 h. Conversely, Dox/P(RGD) NCs exhibited significant cytotoxicity—44% viability following 48 h treatment, which increased substantially over time to 7% and 2% viability after 72 h and 96 h.

Figure 7B exhibits the cell viability following treatment with TRAIL. With free TRAIL, the cytotoxicity was 27% after 48 h and 17% and 8% after 72 h and 96 h. PEGylated TRAIL-conjugated NCs exhibited normal viability after 48 h and only mild toxicity of 51% and 41% after 72 h and 96 h, respectively, while non-PEGylated TRAIL-P(RGD) NCs showed mild toxicity after 48 h (74% viability) and significant toxicity—12% and 9% viability after 72 h and 96 h [52].

The good viability after 48 h of cells treated with PEGylated drug-loaded/conjugated NCs is in accord with the slower drug release due to the slower degradation and penetration. On the other hand, cells treated with Dox, TRAIL, or non-PEGylated drug-bearing NCs responded already after 48 h, with further decline in viability over the next 48 h, showing controlled release in parallel with degradation of the NCs. Treatment with non-PEGylated NCs was found to be more efficient compared with their PEGylated counterparts, showing similar efficiency as free Dox and TRAIL. The results indicate the potential of these NCs in ovarian cancer treatment by decreasing the undesired side effects of Dox as well as the instability of TRAIL while retaining their efficiency, potentially offering an effective dual treatment.

## 4. Summary and Conclusions

This study presented a simple and low-cost synthesis of proteinoid nano-capsules (NCs) based on D-arginine, glycine, and L-aspartic acid building blocks. Self-assembly of the P(RGD) proteinoid in the presence of the wide-spectrum chemotherapeutic drug doxorubicin (Dox), associated with adverse side effects, yielded P(RGD) NCs with Dox encapsulated within the NC core. In addition, tumor necrosis factor-related apoptosis-inducing ligand (TRAIL), which initiates apoptosis in most malignant cells was conjugated for stabilization to the surface of hollow P(RGD) NCs via conjugation to carbodiimide to form TRAIL-P(RGD) NCs. To preserve the integrity of the drugs and allow slow release, PEGylation was done by conjugation to NHS-PEG (3500 MW) maleimide.

Compared with hollow NCs, Dox/P(RGD) NCs exhibited a much smaller dry diameter, while TRAIL-conjugated NCs had similar dry diameters. DLS and Zeta measurements confirmed successful PEGylation and TRAIL conjugation. PEGylated NCs had significantly larger hydrodynamic size, and while negative charges were demonstrated for the hollow and non-PEGylated drug-bearing NCs, the ζ-potential of the PEGylated NCs was elevated, indicating successful conjugation of PEG to surface residues of the NCs. A shift in the excitation peak corresponding to Dox was observed when comparing free Dox to PEGylated and non-PEGylated Dox/P(RGD) NCs (498 vs. 480 nm and 450 nm, respectively), indicating successful encapsulation.

Photostability tests demonstrated that following Dox encapsulation and PEGylation, the fluorescence of Dox/P(RGD) NCs was stable, and even slightly increased for the PEGylated Dox/P(RGD) NCs compared to free Dox. Release of the drug was tested in human serum where, as expected, non-PEGylated Dox/P(RGD) NCs demonstrated improved release.

Cytotoxicity tests in human ovarian cancer cells showed that hollow P(RGD) NCs are non-toxic towards CAOV-3 cells. On the other hand, Dox-encapsulating or TRAIL-conjugated P(RGD) NCs demonstrated toxicity and exhibited controlled release. Non-PEGylated drug-bearing NCs were very effective, with cell viability of 2% and 9% for Dox and TRAIL, respectively, similar to the free drugs. On the other hand, the PEGylated drug-bearing NCs exhibited much lower cytotoxicity—20% and 41% viability for Dox and TRAIL, respectively.

Taken together, these findings confirm the expected role of PEGylation in stabilizing the NCs with less drug leakage, rendering them less effective at acute cancer therapy while allowing slow release over extended periods of time (few days).

Both Dox/P(RGD) and TRAIL-P(RGD) have clear potential for targeted cancer therapy, with potential for dual therapy, combining the reduced side effects of Dox and increased stability of TRAIL. Furthermore, RGD proteinoids may be used to treat other conditions as well.

## Figures and Tables

**Table 1:** Dry and hydrodynamic diameters (nm) of hollow, Dox-loaded and TRAIL-conjugated P(RGD) NCs analyzed by ImageJ software and DLS.

Nanocapsule	Dry Diameter (nm)	Hydrodynamic Diameter (nm)	Zeta Potential (mV)
Hollow P(RGD)	84 ± 16	90 ± 15	−10 ± 7
PEGylated Dox/P(RGD)	46 ± 6	130 ± 17	2 ± 5
Dox/P(RGD)	44 ± 16	118 ± 7	−15 ± 4
PEGylated TRAIL-P(RGD)	90 ± 12	146 ± 16	−7 ± 3
TRAIL-P(RGD)	86 ± 10	134 ± 32	−20 ± 8

**Table 2:** Characteristics of TRAIL-P(RGD) NCs and the Dox/P(RGD) NCs.

Nanocapsule	Initial Drug (w%) ^a^	Entrapment/Conjugation Efficiency (%)
PEGylated Dox/P(RGD)	1.25	62
DOX/P(RGD)	1.25	61
PEGylated TRAIL-P(RGD)	0.05	0.75
TRAIL-P(RGD)	0.05	0.025

^a^ Initial concentration of conjugated or encapsulated TRAIL or Dox.

## Supplementary Materials

The following are available online at https://www.mdpi.com/2073-4360/12/12/2996/s1, Figure S1: FTIR and UV-Vis spectra of P(RGD), Figure S2: Degraded Dox sample chromatogram.
