# Supplementary material for: Engineering of Doxorubicin-Encapsulating and TRAIL-Conjugated Poly(RGD) Proteinoid Nanocapsules for Drug Delivery Applications
Source: Polymers (Basel). 2020 Dec 16;12(12):2996. doi: 10.3390/polym12122996 (PMC7765502; doi:10.3390/polym12122996)
Supplement: Supplementary file 1 [file polymers-12-02996-s001.pdf]

Supplementary material

Engineering of doxorubicin-encapsulating and TRAIL-conjugated poly(RGD) proteinoid nanocapsules for drug delivery applications

Elad Hadad,[a] Safra Rudnick-Glick,[a] Ella Itzhaki,[a] Matan Y. Avivi,[b] Igor Grinberg,[a] Yuval Elias[a] and Shlomo Margel[a]*

[a] Department of Chemistry, Institute of Nanotechnology & Advanced Materials, Bar-Ilan University, Ramat Gan 5290002, Israel

[b] The Mina and Everard Goodman Faculty of Life Sciences, Institute for Nanotechnology and Advanced Materials, Bar Ilan University, Ramat Gan 5290002, Israel

* Correspondence: shlomo.margel@biu.ac.il +972-52-889-8600

Supporting information includes:

Figure S1. FTIR and UV-Vis spectra of P(RGD).

Figure S2. Degraded Dox sample chromatogram.

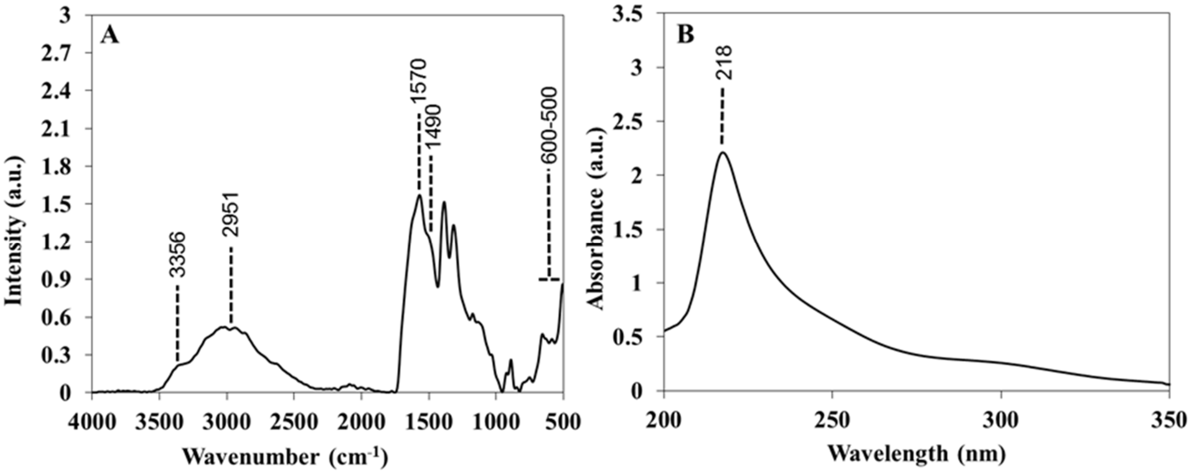

Figure S1. FTIR and UV-Vis spectra of P(RGD). (A) FTIR spectrum (with ATR spectrometer) showing peaks of NH stretching at 2951 and 3356 cm^{-1}, amide CO stretching at 1570 cm^{-1}, amide NH bending at 1490 cm^{-1}, and CO bending at 500–600 cm^{-1}. (B) UV-Vis spectrum showing an absorbance peak at 218 nm, characteristic of peptide bonds.

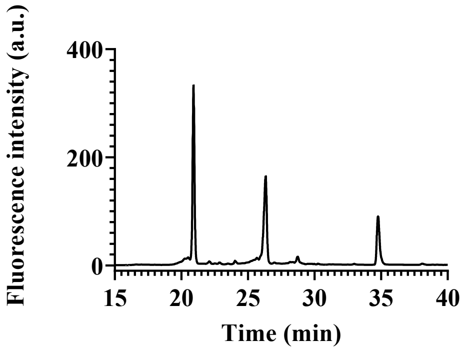

Figure S2. Degraded Dox sample chromatogram. A degraded Dox sample (1 mg/ml) was analyzed during the HPLC method optimization process. Undegraded Dox was detected after 20.9 min, and two degradation products were detected after 26.3 and 34.7 min.
